# Optimization of the Tacrolimus Concentration-to-Dose Ratio Cut-Off Value to Define Metabolism Groups

**DOI:** 10.3390/jcm14082542

**Published:** 2025-04-08

**Authors:** Gerold Thölking, Sophia Hüls, Katharina Schütte-Nütgen, Ulrich Jehn, Hermann Pavenstädt, Stefan Reuter, Raphael Koch

**Affiliations:** 1 Department of Internal Medicine and Nephrology, Herz-Jesu-Hospital Münster-Hiltrup, 48165 Münster-Hiltrup, Germany; 2 Department of Internal Medicine and Nephrology, University Hospital of Münster Marienhospital Steinfurt, 48565 Steinfurt, Germany; sophia.huels@ukm-mhs.de; 3 Department of Medicine D, Division of General Internal Medicine, Nephrology and Rheumatology, University Hospital of Münster, 48149 Münster, Germany; k.schuette-nuetgen@contilia.de (K.S.-N.); jehn@nze-lingen.de (U.J.); hermann.pavenstaedt@ukmuenster.de (H.P.); stefan.reuter@ukmuenster.de (S.R.); 4 Institute of Biostatistics and Clinical Research, University of Münster, 48149 Münster, Germany; koch02@uni-muenster.de

**Keywords:** tacrolimus, renal, kidney, function, survival, transplantation, metabolism, C/D ratio, concentration-to-dose

## Abstract

**Background/Objectives**: The tacrolimus (Tac) concentration-to-dose ratio (C/D ratio) has been described as a predictive marker for several outcome parameters after renal transplantation (RTx). Different C/D ratio values are used to define fast (low C/D ratio) and slow Tac metabolizers (high C/D ratio). In this study, the R package was used to determine the optimal C/D ratio cut-off value to define the Tac metabolism type with a high predictive value for the development of renal function. **Methods**: The data of 389 RTx patients who received an initial immunosuppression with immediate-release tacrolimus (IR-Tac), mycophenolate, prednisolone, and an induction with basiliximab were analyzed. The Tac C/D ratio (ng/mL × 1/mg) of all patients was calculated 3 months after RTx and the maximally selected Wilcoxon statistic was applied to determine the optimal C/D ratio cut-off value for renal function development over a 5-year follow-up. **Results**: A C/D ratio of 0.94 provided the optimal differentiation between fast and slow Tac metabolism in relation to renal function development at 1, 2, 3, and 4 years of follow-up, and at 0.95 five years after RTx. **Conclusions**: As fast Tac metabolism is associated with the development of an impaired renal function, it is essential to identify patients at risk early after RTx. In order to keep the application simple for clinical routine, we suggest calculating the C/D ratio 3 months after RTx and using 1.0 (≤1.0 = fast metabolizer) as the cut-off, which is very close to the optimal value.

## 1. Introduction

Individualized therapy is a significant focus across medical fields [1,2]. In transplant medicine, there is increasing interest in understanding the pharmaco-kinetics, -dynamics, and -genetics to tailor the immunosuppressive treatment, reduce adverse effects, and save costs [3,4]. Especially, under- and over-immunosuppression can have serious consequences, such as acute rejection (AR) or life-threatening infections with consecutive graft impairment or loss. Tacrolimus (Tac) is recommended as the first-line immunosuppressant after renal transplantation (RTx) [5], because of its high efficacy in preventing graft rejection [6]. It is important to know that Tac exhibits high inter- and intra-patient variability in its pharmacokinetics, which poses a significant challenge to maintaining stable therapeutic levels. This variability is largely due to polymorphisms in the cytochrome P450 enzyme system, particularly CYP3A4 and CYP3A5, which influence the metabolism of tacrolimus [7]. In addition, gastrointestinal factors, such as intestinal enzyme expression, diet, and motility, may alter tacrolimus absorption. In addition, extrinsic factors such as concomitant medications, sex, age, and hemoglobin contribute to the pharmacokinetic variability of tacrolimus and require careful therapeutic drug monitoring to avoid under- or over-exposure. However, despite meticulous therapeutic drug monitoring, Tac may cause adverse effects even in patients with Tac trough levels within the intended therapeutic range. In this context, peak Tac levels a few hours after administration are becoming increasingly important as they are associated with the occurrence of calcineurin inhibitor nephrotoxicity (CNIT) [8].

Unfortunately, to date, there is no genetic profile or reliable calculator to determine the individual pre-transplant Tac doses or to calculate the optimal exposure. In recent years, the Tac concentration-to-dose ratio (C/D ratio) has emerged as a promising predictive marker for several outcome parameters after solid organ transplantation [9,10,11]. The Tac C/D ratio can be used as a simple tool to calculate the metabolism rate of Tac [9]. Several groups have demonstrated that a low C/D ratio is associated with poorer outcome parameters after RTx [9,11,12,13,14]. In 2012, Stratta et al. used a weight-adjusted C/D ratio to assess the effect of different clinical covariates on Tac dose requirements in RTx recipients [15]. We have simplified the approach by applying a non-weight-adjusted dose in the C/D ratio formula [8]. In this study, fast Tac metabolism was associated with a reduced renal function. Several studies have confirmed this key finding after RTx [10,12]. Moreover, we and others have observed that fast Tac metabolism is negatively associated with CNIT and rejection [8,9,16,17,18]. These issues lead to a more frequent switch of fast metabolizers to alternative immunosuppressive therapies and, ultimately, result in poorer graft and even patient survival [10,11].

As several groups have suggested different C/D ratio values as being sufficient to detect a difference in renal function between the Tac metabolism groups, statistical methods are required to optimize the cut-off point that best discriminates data in a given cohort based on the observed data. We hypothesize that applying the maximally selected rank statistics method from the R package *maxstat* will allow us to determine an improved C/D ratio cut-off with respect to the estimated glomerular filtration rate (eGFR) outcome.

## 2. Materials and Methods

### 2.1. Study Cohort

This study retrospectively included patients who underwent RTx at the University Hospital Münster between 2007 and 2012. The study was conducted in accordance with the local ethics committee (Ethik-Kommission der Ärztekammer Westfalen-Lippe und der Medizinischen Fakultät der Universität Münster, No. 2014-390-f-N on 17 July 2014) and the ethical principles of the Declaration of Helsinki. The inclusion criteria were an available C/D ratio at 3 months after RTx. The exclusion criteria were any other immunosuppression than IR-Tac (Prograf, Astellas Pharma), mycophenolate, and prednisolone before C/D ratio determination at 3 months, an induction therapy other than basiliximab or thymoglobuline, and graft failure before 3 months after RTx. The IR-Tac blood trough concentrations were used for dose adjustment with a target trough level of 6–10 ng/mL for the first month and 4–7 ng/mL for months 2 and 3. The IR-Tac C/D ratio was calculated by dividing the trough concentration (C) by the corresponding daily dose (D) as published previously [9]. eGFR (CKD-EPI formula [19]) was calculated from the annual creatinine values (years 1–5 after transplantation). Transplantations against donor-specific antibodies were not performed. General patient and recipient data were taken from electronic medical records and data were provided by Eurotransplant. All recorded data were anonymized before analysis. Written informed consent was obtained from all participants before transplantation for the recording and analysis of their clinical data.

### 2.2. Statistics

Statistical analyses were performed using R Statistical Software (version 4.4.2, R Foundation for Statistical Computing, Vienna, Austria) and SAS software, Version 9.4 TS1M8 of the SAS System for Windows (Copyright © 2023 SAS Institute Inc., Cary, NC, USA). All *p*-values and confidence limits were two-sided and intended to be exploratory rather than confirmatory. Consequently, no corrections for multiple comparisons were applied. Exploratory *p*-values of ≤0.05 (two-sided) were regarded as statistically noticeable.

For descriptive statistics, continuous variables with a normal distribution are presented as mean ± standard deviation, whereas non-normally distributed continuous variables are expressed as the median (interquartile range: 25th–75th percentile). Categorical data are summarized as absolute and relative frequencies. To compare metabolism groups, Welch’s *t*-tests were used for normally distributed continuous variables, Mann–Whitney U tests for non-normally distributed continuous data, and Fisher’s exact tests or chi-squared tests for categorical variables. To analyze changes in eGFR within metabolism groups, Wilcoxon signed-rank tests were employed. Box plots were generated to visually display the data.

The optimal cut-off point for the C/D ratio 3 months after RTx regarding the outcome eGFR was determined using the maximally selected Wilcoxon rank statistic as proposed by Hothorn and Lausen [20], implemented in the R package maxstat. The eGFR was assessed at 1, 2, 3, 4, and 5 years after RTx and the cut-off value was determined for each timepoint.

A multivariable linear mixed model was fitted to investigate whether the C/D ratio categorization has an effect on eGFR when adjusting for further covariates, and to model the eGFR course over time, including missing values. The main effects of the covariates European Senior Program (ESP) (yes/no), living donor transplantation (yes/no), age at RTx (years), sex (female/male), diabetes mellitus (yes/no), and the factors time since RTx (years 1, 2, 3, 4, and 5), metabolism group (fast/slow), and the interaction between time and group were included as influencing variables. To account for repeated measurements per patient, a marginal linear mixed model was fitted using SAS PROC MIXED, incorporating an unstructured variance–covariance matrix for residuals, with patients as subjects and time determining the order. The empirical sandwich covariance estimator was applied. Missing data were assumed to be missing at random. Results are reported as least square estimates with a corresponding 95% confidence interval (CI), and *p*-values derived from Wald tests.

Further, we investigated the combined time-to-event point event-free survival (EFS) consisting of the following components: switch from IR-Tac; graft failure; and death as first event (no prior switch or graft failure). Time started at 3 months after RTx as this is when the C/D ratio groups were defined, and all included patients were event-free by month 3. The log-rank test and univariable Cox-regression were used to compare EFS between both groups, and results are reported as hazard ratios (HR) or Kaplan–Meier estimates with pointwise 95% confidence intervals (CI) using log-transformation at 5 years after RTx (4.75 years after metabolism group determination). Additionally, a competing risk analysis was conducted using Fine and Gray’s model, yielding subdistribution hazard ratios (sub-HR) for the components of event-free survival [21]. Cumulative incidence was estimated using the Aalen–Johansen estimator [22]. To compare the cumulative incidence of the respective event types, Gray’s k-sample test was utilized [23]. The cause-specific hazard for each competing event component was assessed between the groups using the method proposed by Prentice [24].

## 3. Results

### 3.1. Patients’ Characteristics

389 RTx recipients were assigned to two metabolism groups according to their 3 months C/D ratio (114 fast and 275 slow metabolizers). There were no noticeable differences between the baseline variables of the enrolled patients at the time of transplantation (Table 1).

### 3.2. C/D Ratio Cut-Off Calculation

**Figure 1:**
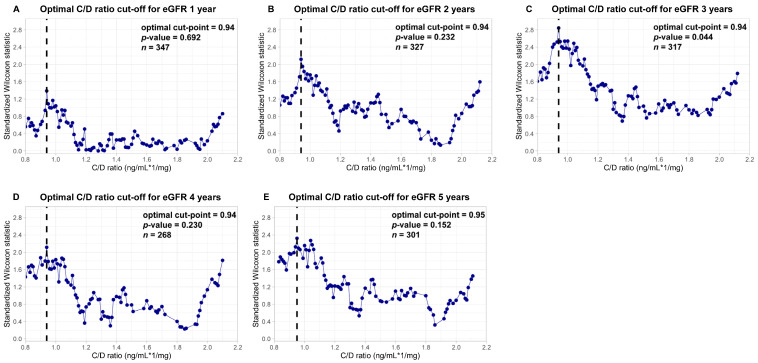
Plots of different C/D ratio cut-off points and standardized Wilcoxon rank statistic for comparison of both groups. The maximally selected Wilcoxon rank statistics from the *maxstat* package in R were used to determine the optimal cut-off points for C/D ratio regarding eGFR at year 1, 2, 3, 4, and 5 after RTx. The exact *p*-values for the maximally selected Gaussian statistics from the test for independence of eGFR and C/D ratio were *p* = 0.692 (year 1), *p* = 0.232 (year 2), *p* = 0.044 (year 3), *p* = 0.230 (year 4), and *p* = 0.152 (year 5). Only in year 3 did the cut-off search result in a statistically noticeable separation of the C/D ratio groups.

A C/D ratio cut-point of 0.94 was found to be optimal for years 1–4 and of 0.95 for year 5 after RTx (Figure 1). The exact *p*-value for the maximally selected Gaussian statistic from the test for independence of eGFR and the C/D ratio was statistically noticeable only at year 3 (*p* = 0.044). Patients were categorized as a fast metabolizer (C/D ratio ≤ 0.94, *n* = 114) or as a slow metabolizer (C/D ratio > 0.94, *n* = 275) based on their 3-month C/D ratio.

### 3.3. Tac Doses, Trough Level, and C/D Ratios

Tac doses were noticeably higher in the fast metabolizer group, while trough levels were lower in this group (Table 2). According to the group definition, the C/D ratio was lower in the fast metabolizers than in the slow metabolizers [0.67 range (0.17–0.94) vs. 1.66 (0.95–6.33) ng/mL·1/mg]. In the group of fast metabolizers, the prednisolone doses [12.5 mg (range 2.5–20)] were similar to those in the slow metabolizers [12.5 mg (range 0–30)] three months after RTx. The dose of mycophenolate mofetil was 1000 (range 500–2000) in both groups.

### 3.4. Renal Function

**Figure 2:**
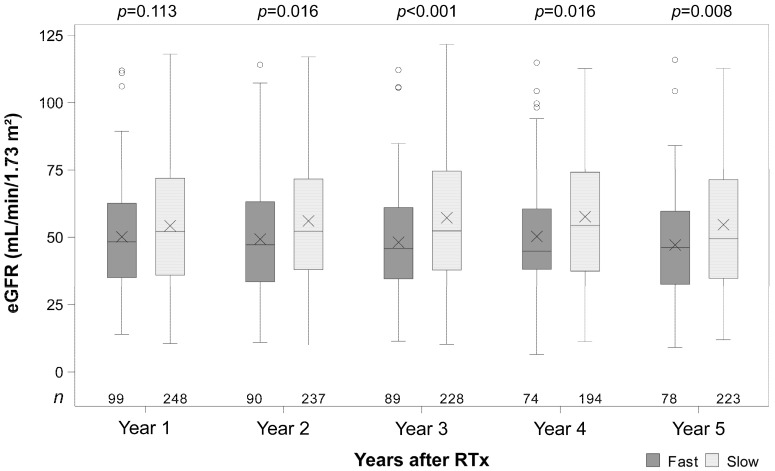
Box plots of the estimated glomerular filtration rates (eGFR) in fast and slow tacrolimus metabolizers. *p*-values are from Welch’s *t*-tests comparing eGFR between fast and slow metabolizers at each time point. X indicates the mean.

**Figure 3:**
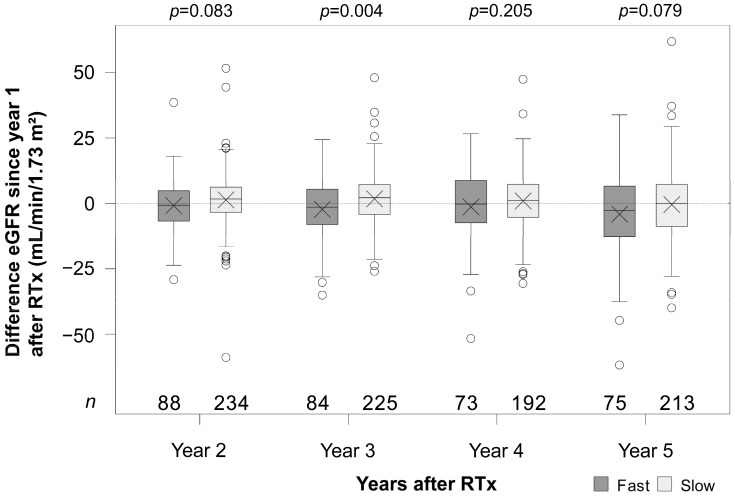
Box plots of the changes in estimated glomerular filtration rates (ΔeGFR) from year 1 after renal transplantation (RTx) in fast and slow tacrolimus metabolizers. *p*-values are from Welch’s *t*-tests comparing ΔeGFR between fast and slow metabolizers at each time point. X indicates the mean.

The renal function of fast Tac metabolizers was on average lower at all time points (1–5 years) and noticeably lower at the time points 2, 3, 4, and 5 years after RTx (all *p* ≤ 0.016, Figure 2). The ΔeGFR (difference between the eGFR of years 2, 3, 4, 5, and year 1) of fast metabolizers was numerically lower at the time points 2, 4, and 5 years and noticeably lower at year 3 (*p* = 0.004, Figure 3). In the multivariable analysis adjusted for further covariates, the mean difference between slow and fast metabolizers was 9.0 mL/min/1.73 m^2^ (95% CI 4.7–13.1) pooled over all time points (Table 3). The groups differed over time (interaction term *p* = 0.039). The mean eGFR decreased slightly in the fast metabolism group, whereas, in slow metabolizers, the mean eGFR was more or less constant over time. Non-ESP transplantation, living donor transplantation, and the female sex were associated with higher eGFR values. In the univariate analysis, the differences between the metabolism groups were similar to those in the multivariable analysis (Figure 2 and Figure 3, Table 3).

### 3.5. Event-Free Survival

**Figure 4:**
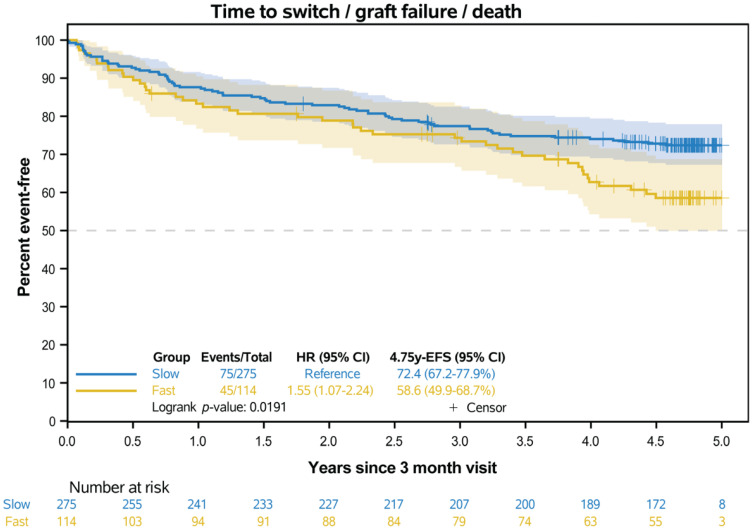
Kaplan–Meier curves for event-free survival in the two metabolism groups. Events were: switch from IR-Tac, graft failure, and death as first event (whichever occurred first). Transparent areas represent the point-wise 95% CIs (log-transformed) of the Kaplan–Meier estimates.

The groups defined by the eGFR optimized cut-off values also differ in EFS (Figure 4). The fast metabolizer had a 1.55 (95% CI 1.07–2.24) times higher hazard of developing an event (Table 4). Competing risk analysis showed that the cumulative incidence of graft failure and death as a first event was more pronounced in fast metabolizers, whereas no difference was observed for switching from IR-Tac as a first event (Table 4).

## 4. Discussion

As different C/D ratio cut-points have been used in RTx studies to differentiate between fast and slow Tac metabolizers, we herein aimed to calculate an optimal cut-point focusing on the endpoint eGFR [10,12,13,18,25,26,27]. On the one hand, the differences between the cut-points (0.86–2.03) used depend on the statistics applied. On the other hand, it must be considered that the C/D ratio depends, to some extent, on the time after transplantation and shows greater uncertainties when early time points are chosen for evaluation [28,29]. Its specific value also depends on the sample size, whether the C/D ratio was based on the mean of the cohort, whether two or three groups were formed, whether an endpoint optimized approach was used, and which endpoint was chosen.

When we started calculating the C/D ratio to define different Tac metabolism groups, we first used a mean C/D ratio at months 1, 3, and 6 and divided our cohort into three nearly similar groups based on the number of patients [9]. This led to the original definition of three groups. C/D ratio < 1.05: fast metabolizer, 1.05–2.03: intermediate metabolizer, and >1.54: slow metabolizer. A comparable approach was later used by others [25,26]. As a next step, we were able to simplify our approach by amending two points. First, the results of the intermediate and slow metabolizers were comparable. Therefore, we decided to proceed with only two groups: fast (C/D ratio < 1.05) and slow metabolizers (C/D ratio ≥ 1.05). The discriminatory potential of the 1.05 cut-off point was later confirmed by Jouve et al. in RTx recipients [11]. However, others have simply split their cohort into two groups and found other cut points to be useful. For example, Nowicka et al. showed that using a C/D ratio cut-off of 1.47 at 6 months after RTx discriminated well between fast and slow metabolizers, with an unfavorable outcome in the fast group [12]. Secondly, the single time point at 3 months for assessment of the C/D ratio seems to be sufficient to determine the Tac metabolic type, as the C/D ratio seems to be relatively stable from this time onwards [10,28]. In addition, we and others showed a good correlation of the C/D ratio three months after RTx to that at 1 and 6 months and 1 month and 1 and 2 years after RTx, resulting in only a small number of misclassifications [10,25]. However, while earlier assessment of the C/D ratio leads to slightly lower cut-off values, as can be seen in the paper by Suwelack et al., a later time point leads to higher cut-off values, as published by Ro et al. [25,26]; a process well-explained by the so-called tacrolimus maturation after RTx [28]. An important reason for this is that Tac metabolism is highly dependent on co-administered drugs [30]. These drug–drug interactions can be of inhibitory or inducing nature. In the early post-transplant period, corticosteroids are the most potent inducers of Tac metabolism [31]. Therefore, the most reasonable time point to calculate the C/D ratio is when corticosteroids are reduced to a minimum.

In addition, anemia, which also plays an important role in the rate of Tac metabolism, improves over time [26]. However, complications, such as infections and acute rejection, often occur within the first few months after RTx, making early risk stratification based on the C/D ratio reasonable [32]. In contrast, Bartlett et al. used a more comprehensive approach and calculated a “final” C/D ratio in a time-weighted, averaging manner at 1, 3, 6, 9, and 12 months after RTx, but found no differences between their two comparison groups [27]. They even observed contradictory results to other studies showing a higher mortality in slow (non-rapid) metabolizers. It should be noted that their cut-off value was 2.03, which is well above the cut-off point published by others. Using this cut-off, the group of fast metabolizers defined in this way would contain a significant number of patients who were actually slow metabolizers, e.g., if our cut-off had been used for classification. Interestingly, the group of fast metabolizers was also three times larger than the group of slow metabolizers, which is the opposite of what we would normally observe in a transplant center with a predominantly Caucasian population [9].

Kwiatkowska et al. used a dichotomized eGFR (<60 and ≥60 mL/min/1.73 m^2^) as an endpoint and performed an AUC analysis to identify the best discriminating C/D ratio cut-off point, which was 1.53 in their cohort. However, the shortcomings of this study are that the timing of the C/D ratio assessment was not standardized and occurred late after RTx (on average 84 months after RTx) and that patients were treated with different tacrolimus formulations [13]. Nevertheless, the authors observed lower eGFR values in patients with a lower C/D ratio or an inverse correlation between concentration and C/D ratio. In principle, this study shows that an eGFR outcome-based calculation of the C/D ratio can be reasonably performed.

The optimal M3 C/D ratio cut-off point for predicting eGFR-based renal outcome in the current study was 0.94 (for the years 1–4) or 0.95 (for year 5). This cut-off point is close to 1.0 and similar to most of the cut-off points described in studies with comparable results [11,33]. To keep the setting simple for a clinical routine, we suggest using the M3 C/D ratio cut-point of 1.0, as this grouping separates the eGFR almost as well (see Figure 1). We additionally performed all calculations for the grouping using the C/D ratio cut-off (≤1.0 vs. >1.0). The results for eGFR and the time-to-event endpoint showed the same trends and differences as for the cut-off of 0.94. Based on this cut-off value, no fast metabolizers are missed and clinicians can identify them easily, namely, when the value of the daily Tac dose is higher than the corresponding trough level.

Our study has several limitations. This is a retrospective study, and, therefore, the interpretation of our data is only hypothesis-generating. The study does not contain any data on the type of previous renal replacement therapy or residual renal function prior to transplantation, which could have had an influence on further renal function. As the C/D ratio or “trough/total daily dose” changes at least until twelve months after transplantation [28,34], we can only conclude from our current data about outcomes calculated on the basis of M3 C/D ratios. The optimal C/D ratio cut-off of 0.94 was statistically noticeable only at 3 years post-transplant, but this threshold was applied to all time points. Future studies should investigate the time-dependent variability in the Tac metabolism. The simplification of rounding the optimal C/D cut-off from 0.94 to 1.0 for clinical convenience requires further validation to ensure predictive accuracy. Our analysis did not account for genetic confounders, such as CYP3A5 polymorphisms, that may influence Tac metabolism. Excluding patients with early graft failure could introduce selection bias by omitting the most severe cases. In addition, the study focused only on IR-Tac, which limits its generalizability to patients on extended-release formulations. Finally, the study relied solely on the C/D ratio as an outcome predictor without incorporating additional biomarkers, such as donor-specific antibodies or donor-derived cell-free DNA, which could provide a more comprehensive risk assessment. Of course, it is important to ensure that the C/D ratio is only assessed in (Tac dose-) stable patients. We recommend that future studies include the information we were unable to account for in this study for a more complete analysis and to validate our proposed C/D ratio threshold.

## 5. Conclusions

Fast Tac metabolizers are at risk of developing adverse outcomes after RTx and should, therefore, be monitored more closely or switched to alternative immunosuppressive therapies. The calculation of an optimized C/D ratio cut-off value of 0.94 carried out here, based on the outcome of the eGFR, can distinguish fast Tac metabolizers from slow Tac metabolizers. By using a M3 C/D ratio cut-point of 1.0 fast metabolizers can be easily identified after RTx.

## Figures and Tables

**Table 1:** Patients’ characteristics.

	Fast Metabolizers (*n* = 114)	Slow Metabolizers (*n* = 275)	*p*-Value
age (years)	50.1 ± 13.7	52.7 ± 13.9	0.099 ^a^
sex (m/f), *n* (%)	60 (52.6%)/54 (47.4%)	167 (60.7%)/108 (39.3%)	0.144 ^b^
BMI (kg/m^2^)	24.8 ± 3.9	25.4 ± 4.1	0.205 ^a^
living donor transplantation	36 (31.6%)	63 (22.9%)	0.096 ^b^
ABO-i	1 (0.9%)	11 (4%)	0.193 ^b^
ESP transplantation	23 (20.2%)	58 (21.1%)	0.892 ^b^
time on waiting list (months)	49 (20–88)	60 (26–91)	0.133 ^c^
DGF	17/113 (16%)	41/274 (16%)	1 ^b^
cold ischemic time (h)	8.2 ± 5.5	8.6 ± 4.9	0.565 ^a^
warm ischemic time (min)	30 (27–35)	30 (28–35)	0.984 ^c^
prior kidney transplantation			
0	99 (86.8%)	234 (85.1%)	0.962 ^b^
1	14 (12.3%)	37 (13.5%)	
2	1 (0.9%)	31 (1.1%)	
3	0	1 (0.4%)	
HLA MM			
0–3	76 (67.3%)	185 (67.5%)	1 ^b^
4–6	37 (32.7%)	89 (32.5%)	
PRA > 20%	2 (1.8%)	6 (2.2%)	1 ^b^
donor characteristics			
donor age (years)	55.0 ± 13.2	51.6 ± 16.5	0.367 ^a^
donor sex (m/f), *n* (%)	49 (43%)/65 (57%)	130 (47.3%)/145 (52.7%)	0.503 ^b^
diagnosis for ESRD			
benign nephrosclerosis	8 (7.0%)	23 (8.4%)	0.516 ^b^
diabetic nephropathy	1 (0.9%)	11 (4.0%)	
polycystic kidney disease	17 (14.9%)	41 (14.9%)	
obstructive nephropathy	11 (9.6%)	22 (8.0%)	
glomerulonephritis	48 (42.1%)	112 (40.7%)	
vasculitis	1 (0.9%)	5 (1.8%)	
interstitial nephritis	1 (0.9%)	5 (1.8%)	
other	27 (23.7%)	55 (20.0%)	
comorbidities before transplantation			
arterial hypertension	108 (95.6%)	260 (94.5%)	0.804 ^b^
diabetes mellitus	12 (10.6%)	37 (13.5%)	0.504 ^b^

Results are reported as frequencies (percentages within group), mean ± standard deviation, or median (25% quantile–75% quantile). *p*-values are from two-sided ^a^ Welch’s *t*-tests, ^b^ Fisher’s exact tests, or ^c^ Mann–Whitney U tests. Abbreviations: BMI, body mass index; ABO-i, ABO incompatible transplantation; ESP, European Senior Program; DGF, delayed graft function; HLA MM, human leucocyte antigen mismatch; PRA, panel reactive antibodies; ESRD, end-stage renal disease.

**Table 2:** Tacrolimus doses, trough levels, and C/D ratios at 3 months after RTx.

	Fast Metabolizers	Slow Metabolizers	*p*-Value
	*n* = 114	*n* = 275	
Tac C/D ratio (ng/mL·1/mg)	0.67 (0.17–0.94)	1.66 (0.95–6.33)	*
Tac dose (mg)	10.0 (7.5–13.0)	5.0 (3.5–6.5)	<0.001
Tac trough levels (ng/mL)	6.6 (4.7–7.9)	8.2 (6.7–10.1)	<0.001

Data presented as mean ± standard deviation or median (minimum–maximum). *p*-values are from Mann–Whitney U tests. * The C/D ratio was used to divide the groups; therefore, differences are trivial. Abbreviations: RTx, renal transplantation; Tac, tacrolimus; C/D, concentration-to-dose.

**Table 3:** Renal function (multivariable linear mixed model).

Model-Based Estimates of eGFR (mL/min/1.73 m^2^)					
Dependent Variables and Contrasts		Estimate	Lower 95% Confidence Limit	Upper 95% Confidence Limit	*p*-Value
ESP transplantation	yes vs. no	−13.3	−19.4	−7.2	<0.001
Living donor transplantation	yes vs. no	6.8	2.13	11.4	0.004
Age at RTx	x vs. x-10 years	−1.8	−3.7	0.4	0.055
Sex	female vs. male	15.0	10.9	19.0	<0.001
Diabetes	yes vs. no	−2.1	−8.9	4.7	0.546
Difference of metabolism over all time points	slow vs. fast	9.0	4.7	13.1	<0.001
Effect of time combined over both metabolism groups					0.004
Interaction term of metabolism groups × time points					0.039
Covariate adjusted least square mean differences between fast and slow metabolizer by time points (combination of main and interaction effects of tacrolimus metabolism group and time points)					
Year 1 after RTx	slow vs. fast	6.1	1.9	10.2	0.004
Year 2 after RTx	slow vs. fast	8.2	3.7	12.7	<0.001
Year 3 after RTx	slow vs. fast	10.3	5.8	14.8	<0.001
Year 4 after RTx	slow vs. fast	9.6	4.8	14.4	<0.001
Year 5 after RTx	slow vs. fast	10.7	5.8	15.5	<0.001
Covariate adjusted least square means of the mean change between the time points (Δ) within metabolism group (combination of main and interaction effects of tacrolimus metabolism group and time points)					
fast metabolizer	Δ year 2 vs. year 1	−1.0	−3.1	1.1	0.358
	Δ year 3 vs. year 1	−2.5	−4.8	−0.1	0.038
	Δ year 4 vs. year 1	−2.6	−5.5	0.4	0.086
	Δ year 5 vs. year 1	−5.2	−8.6	−1.7	0.003
slow metabolizer	Δ year 2 vs. year 1	1.2	−0.2	2.5	0.090
	Δ year 3 vs. year 1	1.8	0.5	3.1	0.007
	Δ year 4 vs. year 1	0.9	−0.6	2.5	0.231
	Δ year 5 vs. year 1	−0.5	−2.3	1.2	0.548
Least square mean differences in the change between time points (Δ) compared between metabolism groups (combination of main and interaction effects of tacrolimus metabolism group and time points)					
Δ year 2 vs. year 1	fast vs. slow	2.1	−0.3	4.6	0.091
Δ year 3 vs. year 1	fast vs. slow	2.1	−0.4	4.6	0.093
Δ year 4 vs. year 1	fast vs. slow	−0.7	−3.0	1.5	0.524
Δ year 5 vs. year 1	fast vs. slow	1.1	−1.4	3.5	0.381

Results of the linear mixed model. Selected parameter estimates and least square means for estimated glomerular filtration rate (eGFR) (mL/min/1.73 m^2^) are shown. Main effects of ESP transplantation, living donor transplantation, age at RTx, sex, diabetes mellitus, metabolism groups, time points, and the interaction term between metabolism group and time were included as influencing factors. *p*-values are from Wald tests. Repeated measurements for each patient were modeled using SAS PROC MIXED by fitting a marginal linear mixed model with an unstructured variance–covariance matrix for the residuals using the empirical sandwich estimator with patient as subject and the order given by time. Abbreviations: RTx, kidney transplantation; ESP, European Senior Program.

**Table 4:** Time-to-event and competing risk analysis.

	Metabolizer Groups		*p*-Value
	Fast (*n* = 114)	Slow (*n* = 275)	
**Event-free survival**			
Number of events, *n*	45	75	-
HR (95% CI)	1.55 (1.07–2.24)	Reference	0.019 *
KM est of EFS at 5 years after RTx, % (95% CI)	59% (50–69)	72% (67–78)	
**Competing risk analysis of event-free survival**			
**Switch from IR-Tac**			
Number of events, *n*	26	53	-
sub-HR (95% CI)	1.22 (0.76–1.94)	Reference	0.406 **
CIF est 5 years after RTx, % (95% CI)	24% (17–33)	19% (15–25)	
cs-HR (95% CI)	1.26 (0.79–2.01)	Reference	0.338 ***
**Graft Failure**			
Number of events, *n*	9	10	-
sub-HR (95% CI)	2.22 (0.90–5.47)	Reference	0.073 **
CIF est 5 years after RTx, % (95% CI)	8.5% (4–16)	4% (2–7)	
cs-HR (95% CI)	2.36 (0.96–5.82)		0.061 ***
**Death (without prior switch or graft failure)**			
Number of events, *n*	10	12	-
sub-HR (95% CI)	2.09 (0.91–4.83)	Reference	0.077 **
CIF est 5 years after RTx, % (95% CI)	9% (5–16.5)	4.5% (3–8)	
cs-HR (95% CI)	2.18 (0.96–5.05)	Reference	0.069 ***

* Comparison of the whole Kaplan–Meier curves between the groups with the log-rank test. ** Gray’s k-sample test for the comparison of the cumulative incidence function for each event component. *** Wald *p*-value from Cox-model with other event types regarded as censored. Cumulative incidence was estimated using the Aalen–Johansen estimator. Cause-specific hazard ratios of all EFS components can only be interpreted together. Abbreviations: EFS, event-free survival; HR, hazard ratio; CI, confidence interval; KM est, Kaplan–Meier estimate; CIF est, cumulative incidence function estimate; sub-HR, subdistribution hazard ratio; cs-HR, cause-specific hazard ratio.

## Data Availability

Data is unavailable due to privacy and ethical restrictions.

## Abbreviations

The following abbreviations are used in this manuscript:

C/D ratio	concentration-to-dose ratio
RTx	renal transplantation
IR-Tac	immediate-release tacrolimus
AR	acute rejection
Tac	tacrolimus
CNI	calcineurin-inhibitor
CNIT	calcineurin inhibitor nephrotoxicity
eGFR	estimated glomerular filtration rate
IQR	interquartile range
CI	confidence interval
ESP	European Senior Program
EFS	event-free survival
HR	hazard ratios
sub-HR	subdistribution hazard ratios
cs-HR	cause-specific hazard
CIF est	cumulative incidence function estimate
BMI	body mass index
ABO-i	ABO incompatible transplantation
DGF	delayed graft function
HLA MM	human leucocyte antigen mismatch
PRA	panel reactive antibodies
ESRD	end-stage renal disease

## References

[1] Evans I.M. (1996). Individualizing therapy, customizing clinical science. J. Behav. Ther. Exp. Psychiatry.

[2] Abubakar M.B., Gan S.H. (2016). Molecular Targets in Advanced Therapeutics of Cancers: The Role of Pharmacogenetics. Oncology.

[3] Andrews L.M., Hesselink D.A., van Schaik R.H.N., van Gelder T., de Fijter J.W., Lloberas N., Elens L., Moes D., de Winter B.C.M. (2019). A population pharmacokinetic model to predict the individual starting dose of tacrolimus in adult renal transplant recipients. Br. J. Clin. Pharmacol..

[4] Yu M., Liu M., Zhang W., Ming Y. (2018). Pharmacokinetics, Pharmacodynamics and Pharmacogenetics of Tacrolimus in Kidney Transplantation. Curr. Drug Metab..

[5] Kidney Disease: Improving Global Outcomes Transplant Work Group (2009). KDIGO clinical practice guideline for the care of kidney transplant recipients. Am. J. Transplant..

[6] Ekberg H., Tedesco-Silva H., Demirbas A., Vitko S., Nashan B., Gurkan A., Margreiter R., Hugo C., Grinyo J.M., Frei U. (2007). Reduced exposure to calcineurin inhibitors in renal transplantation. N. Engl. J. Med..

[7] Oberbauer R., Bestard O., Furian L., Maggiore U., Pascual J., Rostaing L., Budde K. (2020). Optimization of tacrolimus in kidney transplantation: New pharmacokinetic perspectives. Transplant. Rev..

[8] Tholking G., Schutte-Nutgen K., Schmitz J., Rovas A., Dahmen M., Bautz J., Jehn U., Pavenstadt H., Heitplatz B., Van Marck V. (2019). A Low Tacrolimus Concentration/Dose Ratio Increases the Risk for the Development of Acute Calcineurin Inhibitor-Induced Nephrotoxicity. J. Clin. Med..

[9] Tholking G., Fortmann C., Koch R., Gerth H.U., Pabst D., Pavenstadt H., Kabar I., Husing A., Wolters H., Reuter S. (2014). The tacrolimus metabolism rate influences renal function after kidney transplantation. PLoS ONE.

[10] Schutte-Nutgen K., Tholking G., Steinke J., Pavenstadt H., Schmidt R., Suwelack B., Reuter S. (2019). Fast Tac Metabolizers at Risk (-) It is Time for a C/D Ratio Calculation. J. Clin. Med..

[11] Jouve T., Fonrose X., Noble J., Janbon B., Fiard G., Malvezzi P., Stanke-Labesque F., Rostaing L. (2019). The TOMATO study (TacrOlimus MetabolizAtion in kidney TransplantatiOn): Impact of the concentration-dose ratio on death-censored graft survival. Transplantation.

[12] Nowicka M., Gorska M., Nowicka Z., Edyko K., Edyko P., Wislicki S., Zawiasa-Bryszewska A., Strzelczyk J., Matych J., Kurnatowska I. (2019). Tacrolimus: Influence of the Posttransplant Concentration/Dose Ratio on Kidney Graft Function in a Two-Year Follow-Up. Kidney Blood Press. Res..

[13] Kwiatkowska E., Kwiatkowski S., Wahler F., Gryczman M., Domanki L., Marchelk-Mysliwiec M., Ciechanowski K., Drozd-Dabrowska M. (2019). C/D Ratio in Long-Term Renal Function. Transplant. Proc..

[14] Debska-Slizien A., Kuzmiuk-Glembin I., Hozejowski R., Kaminska D., Krajewska M., Zawiasa-Bryszewska A., Kurnatowska I., Smykal-Jankowiak K., Glyda M., Koziol L. (2024). Renal Allograft Function and the Tacrolimus C/D Ratio: Insights from a Prospective Study on MeltDose Tacrolimus. J. Clin. Med..

[15] Stratta P., Quaglia M., Cena T., Antoniotti R., Fenoglio R., Menegotto A., Ferrante D., Genazzani A., Terrazzino S., Magnani C. (2012). The interactions of age, sex, body mass index, genetics, and steroid weight-based doses on tacrolimus dosing requirement after adult kidney transplantation. Eur. J. Clin. Pharmacol..

[16] Kuypers D.R., Naesens M., de Jonge H., Lerut E., Verbeke K., Vanrenterghem Y. (2010). Tacrolimus dose requirements and CYP3A5 genotype and the development of calcineurin inhibitor-associated nephrotoxicity in renal allograft recipients. Ther. Drug Monit..

[17] Egeland E.J., Reisaeter A.V., Robertsen I., Midtvedt K., Strom E.H., Holdaas H., Hartmann A., Asberg A. (2019). High tacrolimus clearance—A risk factor for development of interstitial fibrosis and tubular atrophy in the transplanted kidney: A retrospective single-center cohort study. Transpl. Int..

[18] Egeland E.J., Robertsen I., Hermann M., Midtvedt K., Storset E., Gustavsen M.T., Reisaeter A.V., Klaasen R., Bergan S., Holdaas H. (2017). High Tacrolimus Clearance Is a Risk Factor for Acute Rejection in the Early Phase After Renal Transplantation. Transplantation.

[19] Levey A.S., Stevens L.A., Schmid C.H., Zhang Y.L., Castro A.F., Feldman H.I., Kusek J.W., Eggers P., Van Lente F., Greene T. (2009). A new equation to estimate glomerular filtration rate. Ann. Intern. Med..

[20] Hothorn T., Lausen B. (2003). On the exact distribution of maximally selected rank statistics. Comput. Stat. Data Anal..

[21] Fine J.P., Gray R.J. (1999). A Proportional Hazards Model for the Subdistribution of a Competing Risk. J. Am. Stat. Assoc..

[22] Aalen O.O., Johansen S. (1978). An Empirical Transition Matrix for Non-Homogeneous Markov Chains Based on Censored Observations. Scand. J. Stat..

[23] Gray R.J. (1988). A Class of K-Sample Tests for Comparing the Cumulative Incidence of a Competing Risk. Ann. Stat..

[24] Prentice R.L., Kalbfleisch J.D., Peterson A.V., Flournoy N., Farewell V.T., Breslow N.E. (1978). The Analysis of Failure Times in the Presence of Competing Risks. Biometrics.

[25] Suwelack B., Bunnapradist S., Meier-Kriesche U., Stevens D.R., Procaccianti C., Morganti R., Budde K. (2020). Effect of Concentration/Dose Ratio in De Novo Kidney Transplant Recipients Receiving LCP-Tacrolimus or Immediate-Release Tacrolimus: Post Hoc Analysis of a Phase 3 Clinical Trial. Ann. Transplant..

[26] Ro H., Jeong J.C., Kong J.M., Min J.W., Park S.K., Lee J., Koo T.Y., Yang J., Kim M.S., Hwang S. (2021). The tacrolimus metabolism affect post-transplant outcome mediating acute rejection and delayed graft function: Analysis from Korean Organ Transplantation Registry data. Transpl. Int..

[27] Bartlett F.E., Carthon C.E., Hagopian J.C., Horwedel T.A., January S.E., Malone A. (2019). Tacrolimus Concentration-to-Dose Ratios in Kidney Transplant Recipients and Relationship to Clinical Outcomes. Pharmacotherapy.

[28] Rostaing L., Bunnapradist S., Grinyo J.M., Ciechanowski K., Denny J.E., Silva H.T., Budde K., Kulkarni S., Hricik D., Bresnahan B.A. (2016). Novel Once-Daily Extended-Release Tacrolimus Versus Twice-Daily Tacrolimus in De Novo Kidney Transplant Recipients: Two-Year Results of Phase 3, Double-Blind, Randomized Trial. Am. J. Kidney Dis..

[29] von Samson-Himmelstjerna F.A., Messtorff M.L., Kakavand N., Eisenberger U., Korth J., Lange U., Kolbrink B., Aldag L., Schulze Dieckhoff T., Feldkamp T. (2023). The Tacrolimus Concentration/Dose Ratio Does Not Predict Early Complications After Kidney Transplantation. Transpl. Int..

[30] Lemaitre F., Budde K., Van Gelder T., Bergan S., Lawson R., Noceti O., Venkataramanan R., Elens L., Moes D., Hesselink D.A. (2022). Therapeutic drug monitoring and dosage adjustments of immunosuppressive drugs when combined with nirmatrelvir/ritonavir in patients with COVID-19. Ther. Drug Monit..

[31] Anglicheau D., Flamant M., Schlageter M.H., Martinez F., Cassinat B., Beaune P., Legendre C., Thervet E. (2003). Pharmacokinetic interaction between corticosteroids and tacrolimus after renal transplantation. Nephrol. Dial. Transplant..

[32] Sellares J., de Freitas D.G., Mengel M., Reeve J., Einecke G., Sis B., Hidalgo L.G., Famulski K., Matas A., Halloran P.F. (2012). Understanding the causes of kidney transplant failure: The dominant role of antibody-mediated rejection and nonadherence. Am. J. Transplant..

[33] Tomizawa M., Hori S., Inoue K., Nishimura N., Nakai Y., Miyake M., Yoneda T., Fujimoto K. (2023). A Low Tacrolimus Concentration-to-Dose Ratio Increases Calcineurin Inhibitor Nephrotoxicity and Cytomegalovirus Infection Risks in Kidney Transplant Recipients: A Single-Center Study in Japan. Transplant. Proc..

[34] Kwiatkowska E., Ciechanowski K., Domanski L., Dziedziejko V., Przybycinski J., Pawlik A. (2022). Intrapatient Variability (IPV) and the Blood Concentration Normalized by the Dose (C/D Ratio) of Tacrolimus-Their Correlations and Effects on Long-Term Renal Allograft Function. Biomedicines.

